# Transurethral surgical treatment for benign prostatic hyperplasia with detrusor underactivity: a systematic review and meta-analysis

**DOI:** 10.1186/s13643-024-02514-3

**Published:** 2024-03-22

**Authors:** Peilin Zou, Chang Liu, Yucong Zhang, Chao Wei, Xiaming Liu, Shengfei Xu, Qing Ling, Zhong Chen, Guanghui Du, Xiaoyi Yuan

**Affiliations:** 1 Department of Geriatrics, Tongji Hospital, Tongji Medical College, Huazhong University of Science and Technology, Wuhan, Hubei 430030 China; 2 Key Laboratory of Vascular Aging, Ministry of Education, Tongji Hospital, Tongji Medical College, Huazhong University of Science and Technology, 1095 Jiefang Avenue, Wuhan, 430030 China; 3 The Second Clinical School, Tongji Hospital, Tongji Medical College, Huazhong University of Science and Technology, Wuhan, China; 4 Department of Urology, Tongji Hospital, Tongji Medical College, Huazhong University of Science and Technology, Wuhan, China

**Keywords:** Transurethral surgical treatment, Detrusor underactivity, Systematic review, Meta-analysis

## Abstract

**Background:**

The efficacy of surgical treatment for benign prostatic hyperplasia (BPH) patients with detrusor underactivity (DU) remains controversial.

**Methods:**

To summarize relevant evidence, three databases (PubMed, Embase, and Web of Science) were searched from database inception to May 1, 2023. Transurethral surgical treatment modalities include transurethral prostatectomy (TURP), photoselective vaporization of the prostate (PVP), and transurethral incision of the prostate (TUIP). The efficacy of the transurethral surgical treatment was assessed according to maximal flow rate on uroflowmetry (*Q*_max_), International Prostate Symptom Score (IPSS), postvoid residual (PVR), quality of life (QoL), voided volume, bladder contractility index (BCI) and maximal detrusor pressure at maximal flow rate (PdetQ_max_). Pooled mean differences (MDs) were used as summary statistics for comparison. The quality of enrolled studies was evaluated by using the Newcastle–Ottawa Scale. Sensitivity analysis and funnel plots were applied to assess possible biases.

**Results:**

In this study, 10 studies with a total of 1142 patients enrolled. In BPH patients with DU, within half a year, significant improvements in *Q*_max_ (pooled MD, 4.79; 95% CI, 2.43–7.16; *P* < 0.05), IPSS(pooled MD, − 14.29; 95%CI, − 16.67–11.90; *P* < 0.05), QoL (pooled MD, − 1.57; 95% CI, − 2.37–0.78; *P* < 0.05), voided volume (pooled MD, 62.19; 95% CI, 17.91–106.48; *P* < 0.05), BCI (pooled MD, 23.59; 95% CI, 8.15–39.04; *P* < 0.05), and PdetQ_max_ (pooled MD, 28.62; 95% CI, 6.72–50.52; *P* < 0.05) were observed after surgery. In addition, after more than 1 year, significant improvements were observed in *Q*_max_ (pooled MD, 6.75; 95%CI, 4.35–9.15; *P* < 0.05), IPSS(pooled MD, − 13.76; 95%CI, − 15.17–12.35; *P* < 0.05), PVR (pooled MD, − 179.78; 95%CI, − 185.12–174.44; *P* < 0.05), QoL (pooled MD, − 2.61; 95%CI, − 3.12–2.09; *P* < 0.05), and PdetQ_max_ (pooled MD, 27.94; 95%CI, 11.70–44.19; *P* < 0.05). Compared with DU patients who did not receive surgery, DU patients who received surgery showed better improvement in PVR (pooled MD, 137.00; 95%CI, 6.90–267.10; *P* < 0.05) and PdetQ_max_ (pooled MD, − 8.00; 95%CI, − 14.68–1.32; *P* < 0.05).

**Conclusions:**

Our meta-analysis results showed that transurethral surgery can improve the symptoms of BPH patients with DU. Surgery also showed advantages over pharmacological treatment for BPH patients with DU.

**Systematic review registration:**

PROSPERO CRD42023415188.

**Supplementary Information:**

The online version contains supplementary material available at 10.1186/s13643-024-02514-3.

## Introduction

Voiding is influenced by bladder detrusor contraction and urethral patency. Detrusor underactivity (DU) is a common lower urinary tract dysfunction that typically presents as structural or functional abnormalities of the urinary tract and its surrounding tissues [1]. It commonly results in incomplete bladder emptying and other troublesome lower urinary tract symptoms (LUTS). At the same time, lower urinary tract obstruction due to benign prostatic hyperplasia (BPH) can also affect voiding. It is reported that the prevalence of DU in men with LUTS is about 9–48%, and BPH is present in approximately 8% of men in the fourth decade of life but up to 90% of men in the ninth decade [2, 3]. This proportion is constantly rising. It has a detrimental influence on patients' health and quality of life and needs to be well managed.

Currently, the treatment modalities for BPH patients with DU include pharmacological and surgical modalities. However, the efficacy of pharmacological treatment is unsatisfactory, and the efficacy of surgical treatment for BPH patients with DU remains controversial, especially for transurethral surgical treatment, although some studies have reported the efficacy of surgery in men with BPH and DU [4, 5]. As a result, there is an urgent need to summarize the findings of relevant researches.

Transurethral surgical treatment mainly includes transurethral prostatectomy (TURP), photoselective vaporization of the prostate (PVP), holmium laser enucleation of the prostate (HoLEP), transurethral incision of the prostate (TUIP). These surgical modalities have long been considered the gold standard for surgical treatment of BPH [6].

The aim of this research is to conduct a systematic review and meta-analysis of published literature regarding the effect of transurethral surgical treatment on BPH patients with DU.

## Materials and methods

### Search strategy

The protocol for this systematic review was developed prospectively and registered in PROSPERO (CRD42023415188). The systematic review was reported following the Preferred Reporting Items for MOOSE and PRISMA recommendations [7, 8]. A comprehensive online literature search using the following search terms was performed: PubMed, Web of Science, and Embase (via Elsevier). The search query was as follows: (“underactive bladder” OR “detrusor underactivity”) AND (“surgery” OR “surgical treatment” OR “prostatic artery embolization”). The article search was performed in May 2023.

### Selection of eligible studies

The inclusion criteria included are as follows: (1) articles published in English; (2) articles regarding BPH patients with DU who underwent transurethral surgical treatment or not; (3) articles compared maximal flow rate on uroflowmetry (*Q*_max_), International Prostate Symptom Score (IPSS), postvoid residual (PVR), quality of life (QoL), voided volume, bladder contractility index (BCI) and maximal detrusor pressure at maximal flow rate (PdetQ_max_.) (at least one parameter); (4) articles reported definite sample size. When duplication of patient data was suspected, the earliest published article was selected. If eligible data were not available in the article that met the inclusion criteria, we contacted the corresponding author by email to obtain the needed data. Review papers, letters, preclinical studies, or articles with insufficient information were excluded by screening. Two review authors (PZ and CL) screened the search results, first in title and abstract, and subsequently in full text.

### Data acquisition and quality assessment

**Fig. 1:**
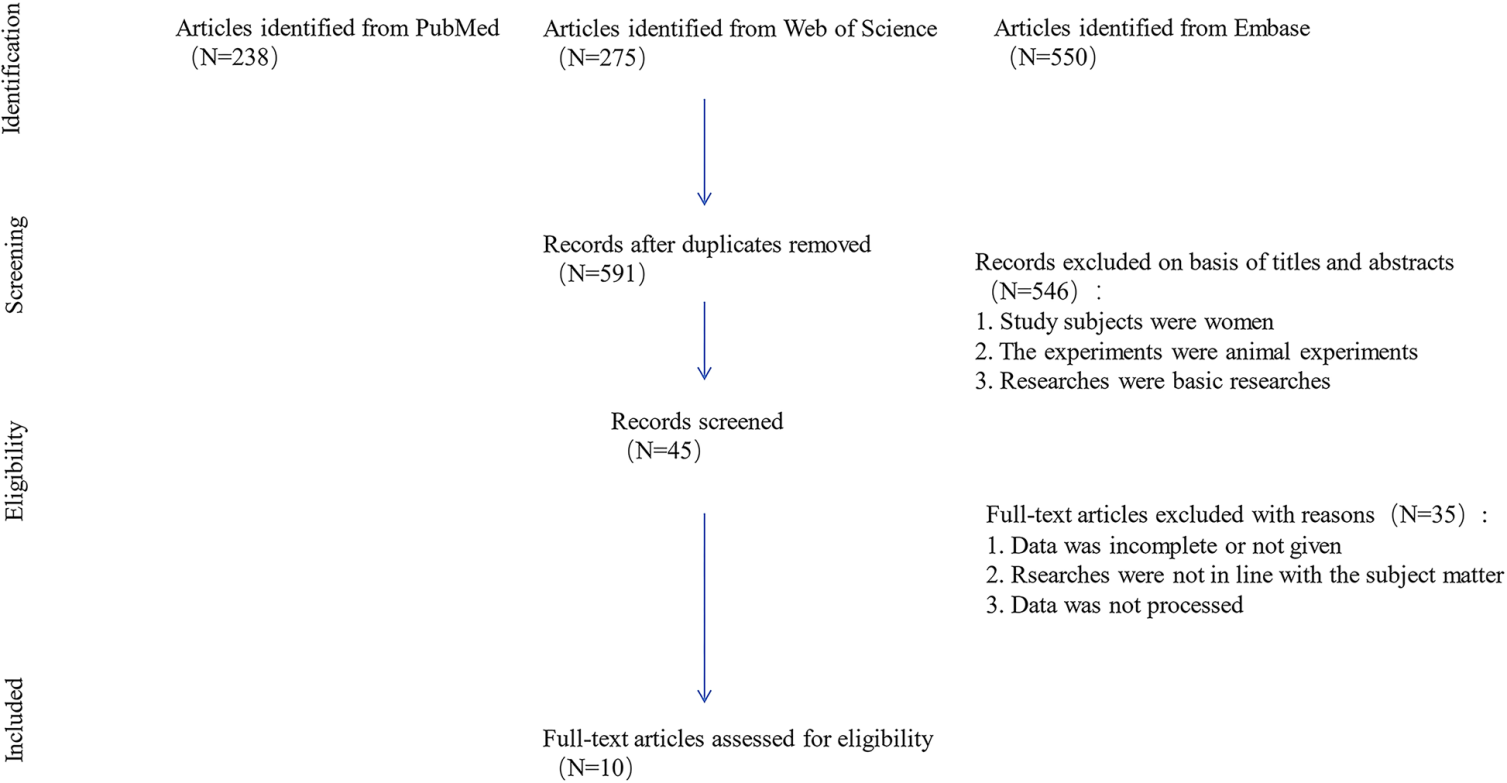
Flow diagram for studies included in and excluded from the meta-analysis

Population size, number of each subgroup by the DU degree of preoperative, and mean improvement of maximal flow rate on uroflowmetry (*Q*_max_), International Prostate Symptom Score (IPSS), postvoid residual (PVR), quality of life (QoL), voided volume, bladder contractility index (BCI) and maximal detrusor pressure at maximal flow rate (PdetQ_max_.) of each subgroup with standard deviation (SD) were retrieved for data synthesis. Figure 1 provides information on the data analysis procedure in more detail. Because all of the compared outcome parameters were continuous variables, pooled mean differences (MDs) were used as summary statistics for comparison. Data acquisition was performed by two independent reviewers (YZ and WC). The quality of qualifying studies was evaluated by using the Newcastle–Ottawa Scale for cohort studies criteria, which has a maximum total score of 9 based on the assessment of three domains: (1) selection of study groups, (2) comparability of groups, and (3) ascertainment of the outcome of interest. Studies with a total score of 1 to 3, 4 to 6, and 7 to 9 on the NOS scale were considered low, intermedia, and high quality, respectively [9]. Two review authors (XL and SX) conducted quality assessment independently.

### Data analysis

Review Manager Software version 5.4.1 was used to calculate the effect sizes. A random-effects model and fixed-effects model were adopted to obtain the pooled MDs and 95% confidence intervals (CIs). Heterogeneity was tested by using the chi-squared test and *I*^2^ statistic. *p* < 0.05 or *I*^2^ > 50% indicated that the heterogeneity was significant. The overall effects were determined by the *Z*-test, and *p* < 0.05 was considered statistically significant.

### Assessments of possible biases

Stata17 was used to lead a sensitivity analysis to discover whether the effects of diagnostic threshold, study population characteristics, or surgery type are present in the final result. For each comparison, funnel plots were applied to examine the potential for publication bias. If the funnel plots were not symmetrical, Egger’s regression test using stata17 was used for the outcome.

### Ethics statement

The present study protocol was reviewed and approved by PROSPERO (https://www.crd.york.ac.uk/PROSPERO/. (Reg. No. CRD42023415188).

## Results

### Search result

Database searches identified 1063 references (PubMed 238; Embase 550; Web of Science 275). After deduplication, 591 references were screened in the title/abstract, and 546 were excluded. Forty-five articles were screened in full text. Thirty-five were excluded for several reasons (Fig. 1). Ultimately, 10 studies were included in the meta-analysis with 1142 patients enrolled (Table 1) [10–19].

**Table 1:** Description and characteristics of the eligible studies

Study	Year	Country	Study design	Total study population	Type of surgery	Time of outcome evaluation (month)	Quality assessment^a^	Comparator
Thomas	2004	UK	Retrospective cohort	84	TURP	135.6 (mean)	7	Without surgery
Masumori	2010	Japan	Retrospective self-controlled	12	TURP	3, 36, 84	7	Before surgery
Choi	2011	Korea	Retrospective self-controlled	371	PVP	1, 12	8	Before surgery
Yu	2015	China	Retrospective self-controlled	78	PVP	12	8	Before surgery
Sokhal	2017	India	Retrospective self-controlled	174	TURP	3	8	Before surgery
Lee	2019	China	Retrospective self-controlled	60	TURP/TUIP	3	8	Before surgery
Thomas	2019	USA	Retrospective self-controlled	106	PVP	1, 3, 6, 12	8	Before surgery
Rubilotta	2020	Italy	Prospective self-controlled	51	TURP	More than 24 (mean)	9	Before surgery
Wu	2020	China	Retrospective self-controlled	48	TURP/TUIP	24.9 (mean)	8	Before surgery
Lebani	2023	Brazil	Prospective self-controlled	158	TURP	1,6,12	9	Before surgery

^a^Evaluated using Newcastle–Ottawa Scale for cohort studies

### General characteristics of included studies

The general characteristics of the included studies are shown in Table 2. Among 10 studies, eight studies [11, 14] were retrospective, and the rest were prospective studies [10, 12, 13, 15–19]. The median follow-up time of studies was 36 months, ranging from 1 month to 7 years. All studies compared the improvement of urodynamic examination data of BPH patients with DU after transurethral surgical treatment. One study compared the difference between urodynamic examination data of patients who underwent transurethral surgical treatment and not. Some of the enrolled patients in the two studies included DU patients without a diagnosis of BPH. According to the NOS scales, all included studies were of high quality (Supplementary Table 1).

**Table 2:** Patient characteristics

Study		Compared outcome parameters						
		*Q*_max_	IPSS	PVR	QoL score	Voided volume	BCI	PdetQ_max_
Thomas	2004	Available	NA	Available	NA	Available	Available	Available
Masumori	2010	NA	Available	NA	Available	NA	NA	NA
Choi	2011	NA	Available	NA	NA	NA	NA	NA
Yu	2015	Available	Available	Available	Available	NA	NA	Available
Sokhal	2017	Available	Available	Available	Available	NA	Available	Available
Lee	2019	Available	NA	Available	NA	Available	Available	Available
Thomas	2019	Available	Available	NA	Available	NA	NA	Available
Rubilotta	2020	Available	Available	Available	NA	NA	NA	NA
Wu	2020	Available	NA	Available	NA	NA	Available	Available
Lebani	2023	Available	NA	NA	NA	NA	NA	NA

### Differences in parameters before and after surgery

**Fig. 2:**
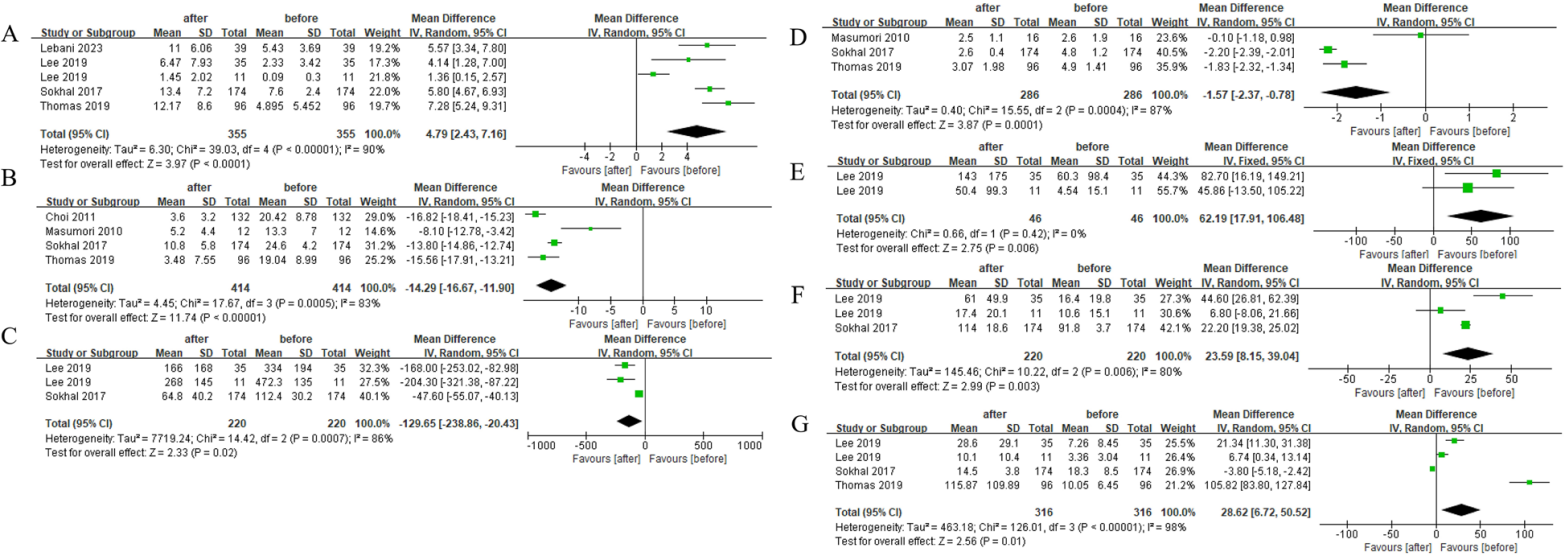
The comparison of each value of DU patients before and after surgery within half a year. **A** *Q*_max_, maximal flow rate on uroflowmetry. **B** IPSS, International Prostate Symptom Score. **C** PVR, post-void residual. **D** QoL, quality of life. **E** Voided volume. **F** BCI, bladder contractility index. **G** PdetQ_max_, maximal detrusor pressure at maximal flow rate

**Fig. 3:**
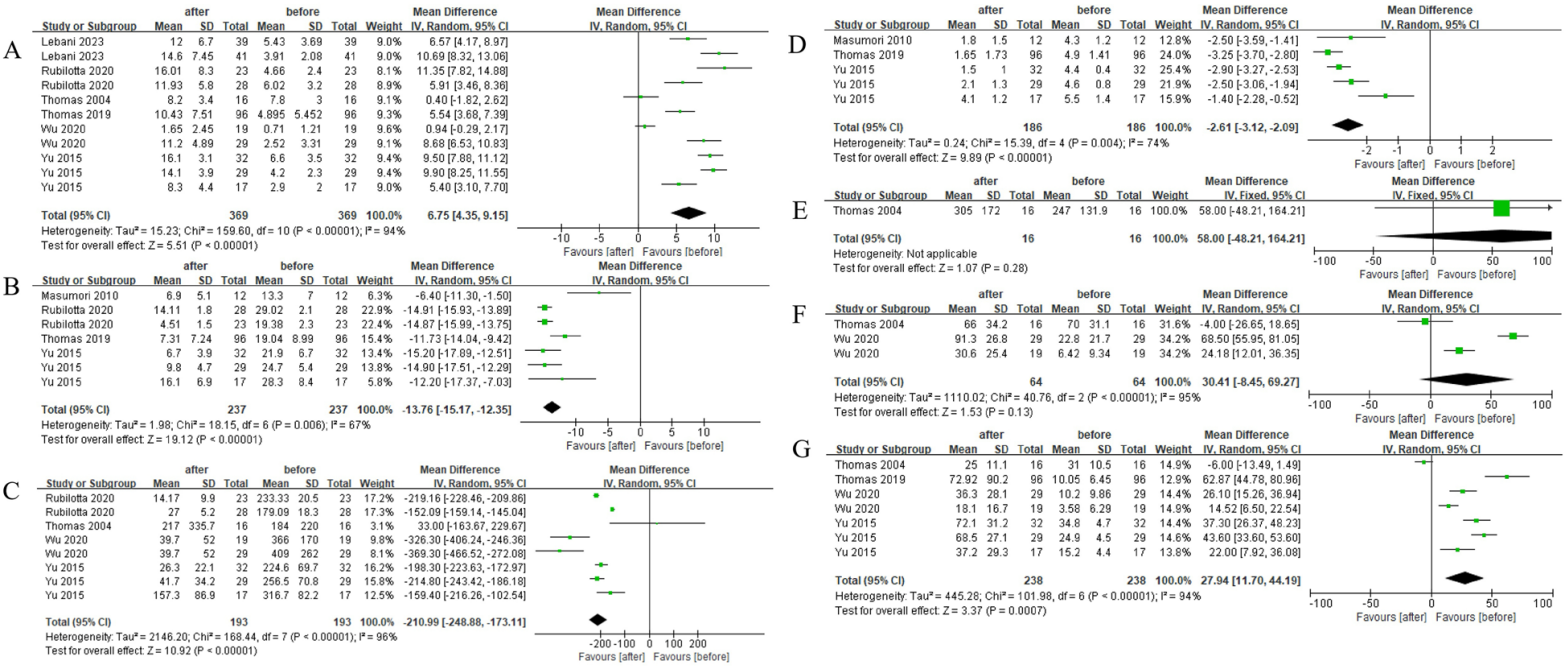
The comparison of each value of DU patients before and after surgery for more than a year. **A** *Q*_max_, maximal flow rate on uroflowmetry. **B** IPSS, International Prostate Symptom Score. **C** PVR, post-void residual. **D** QoL, quality of life. **E** Voided volume. **F** BCI, bladder contractility index. **G** PdetQ_max_, maximal detrusor pressure at maximal flow rate

Forest plots comparing the improvements in outcome parameters between preoperative and postoperative data are shown in Figs. 2 and 3.

The *Q*_max_ (pooled MD, 4.79; 95% CI, 2.43–7.16; Fig. 2B), voided volume (pooled MD, 62.19; 95% CI, 17.91–106.48; Fig. 2E), BCI (pooled MD, 23.59; 95% CI, 8.15–39.04; Fig. 2F) and PdetQ_max_ (pooled MD, 28.62; 95% CI, 6.72–50.52; Fig. 2G) were significantly elevated after surgery within half a year. Meanwhile, the IPSS (pooled MD, − 14.29; 95% CI, − 16.67 to − 11.90; Fig. 2B), PVR (pooled MD, − 129.65; 95% CI, − 238.86–20.43; Fig. 2C), and QoL (pooled MD, − 1.57; 95% CI, − 2.37–0.78; Fig. 2D) were significantly decreased after surgery.

More than a year after surgery, *Q*_max_ (pooled MD, 6.75; 95% CI, 4.35–9.15; Fig. 3A), BCI (pooled MD, 39.22; 95% CI, 31.07–47.38; Fig. 3F), and PdetQ_max_ (pooled MD, 27.94; 95% CI, 11.70–44.19; Fig. 3G) were significantly elevated, and IPSS (pooled MD, − 13.76; 95% CI, − 15.17 to − 12.35; Fig. 2B), PVR (pooled MD, − 179.78; 95% CI, − 185.12 to − 174.44; Fig. 3C), and QoL (pooled MD, − 2.61; 95% CI, − 3.12 to − 2.09; Fig. 3D) were significantly decreased. However, there was no significant change in voided volume (pooled MD, 58; 95% CI, − 48.21–164.21; Fig. 3E).

### Differences of parameters in patients received surgery or not

**Fig. 4:**
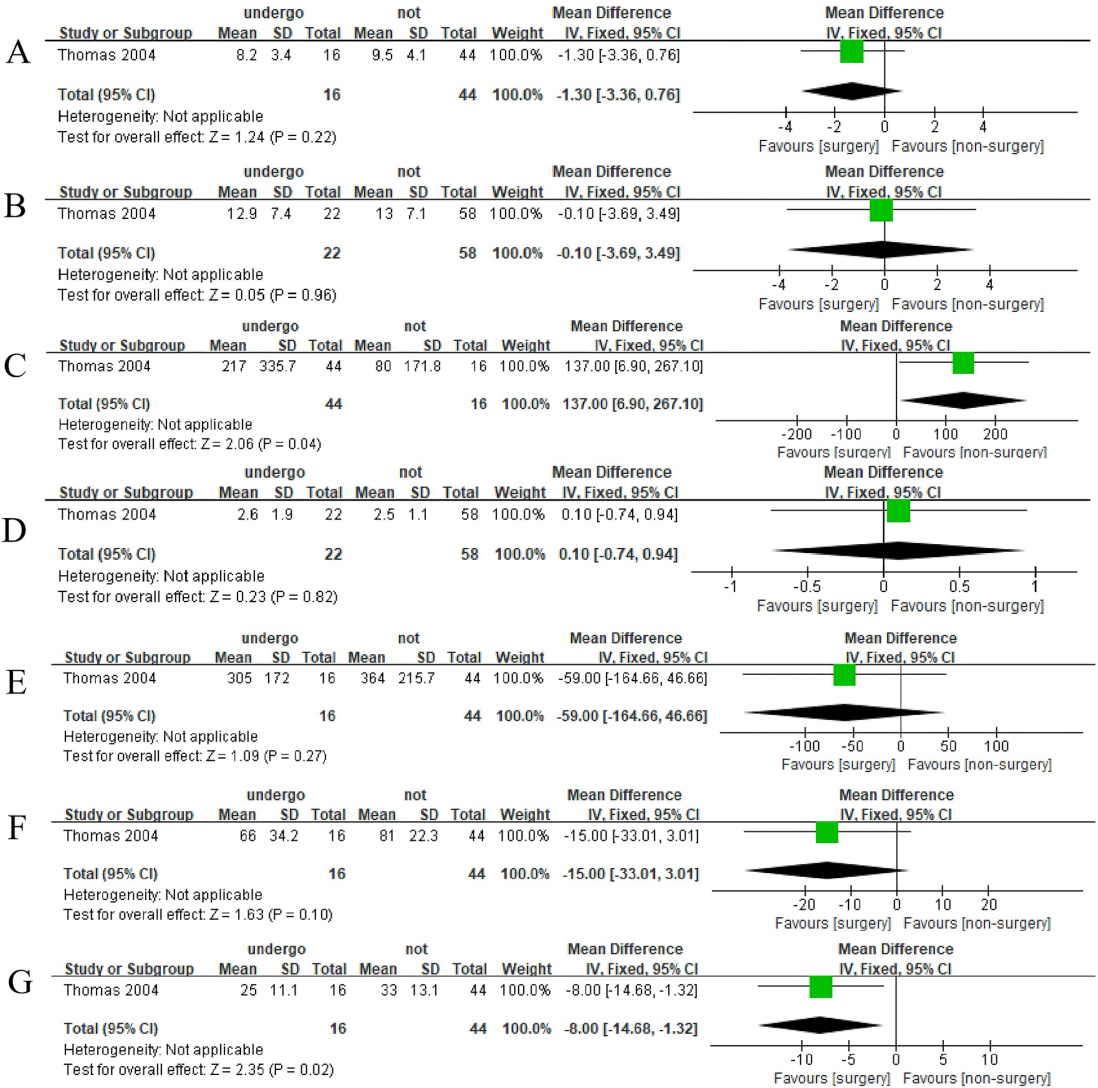
The comparison of each value of DU patients undergoing surgery or not with 135.6 months follow-up. A *Q*_max_, maximal flow rate on uroflowmetry. **B** IPSS, International Prostate Symptom Score. **C** PVR, post-void residual. **D** QoL, quality of life. **E** Voided volume. **F** BCI, bladder contractility index. **G** PdetQ_max_, maximal detrusor pressure at a maximal flow rate

Compared with patients who did not receive surgery, the PVR (pooled MD, 137.00; 95% CI, 6.90–267.10; Fig. 4C) was higher in patients who underwent surgery and PdetQ_max_ (pooled MD, − 8.00; 95% CI, − 14.68–1.32; Fig. 4G) was lower. However, the differences between *Q*_max_ (pooled MD, − 1.30; 95%CI, − 3.36–0.76; Fig. 4A), IPSS (pooled MD, − 0.10; 95%CI, − 3.69–3.49; Fig. 3B), QoL (pooled MD, 0.10; 95%CI, − 0.74–0.94; Fig. 4D), voided volume (pooled MD, − 59; 95%CI, − 164.66–46.66; Fig. 4E), BCI (pooled MD, -15.00; 95%CI, − 33.01–3.01; Fig. 4F) between patients received surgery or not.

### Result of sensitivity analysis

**Fig. 5:**
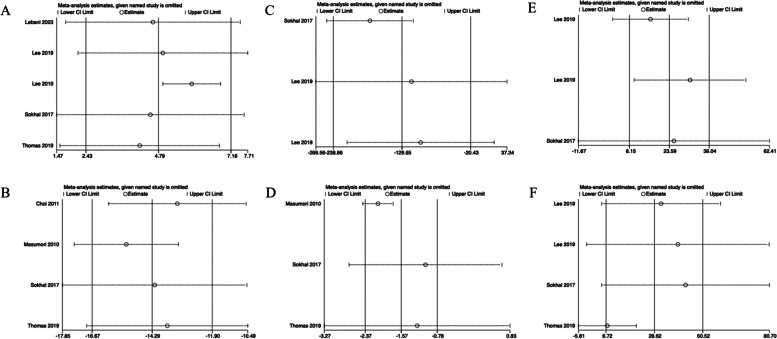
The sensitivity analysis of each value of DU patients before and after surgery within half a year. **A** *Q*_max_, maximal flow rate on uroflowmetry. **B** IPSS, International Prostate Symptom Score. **C** PVR, post-void residual. **D** QoL, quality of life. **E** BCI, bladder contractility index. **F** PdetQ_max_, maximal detrusor pressure at a maximal flow rate

**Fig. 6:**
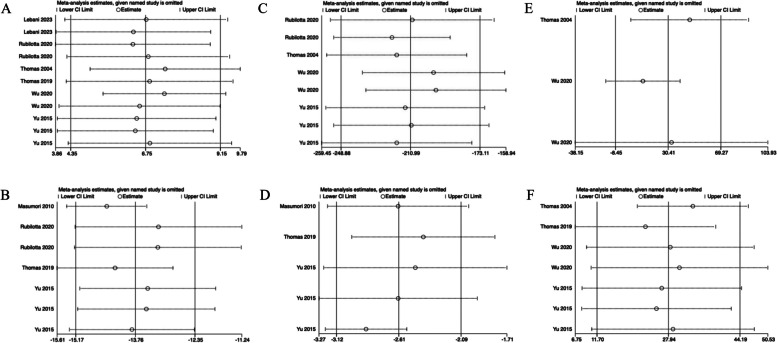
The sensitivity analysis of each value of DU patients before and after surgery more than a year. **A** *Q*_max_, maximal flow rate on uroflowmetry. **B** IPSS, International Prostate Symptom Score. **C** PVR, post-void residual. **D** QoL, quality of life; E. BCI, bladder contractility index. **F** PdetQ_max_, maximal detrusor pressure at maximal flow rate

The results for PVR, QoL, BCI, and PdetQ_max_ showed instability (Figs. 5 and 6), which may be caused by the difference in surgical modalities used for patients. In data after more than a year, only the result for BCI shows instability, which indicates that the index after 1 year is more stable. The reason for instability may be the differences in the classification criteria of DU patients.

### Assessment of heterogeneity and publication bias

**Fig. 7:**
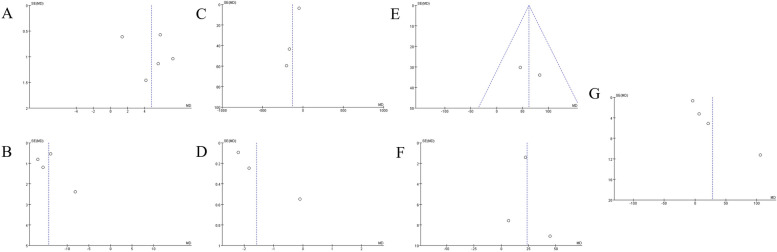
The funnel plot of each value of DU patients before and after surgery within half a year. **A** Q_max_, maximal flow rate on uroflowmetry. **B** IPSS, International Prostate Symptom Score. **C** PVR, post-void residual. **D** QoL, quality of life. **E** voided volume. **F** BCI, bladder contractility index. **G** PdetQ_max_, maximal detrusor pressure at maximal flow rate

**Fig. 8:**
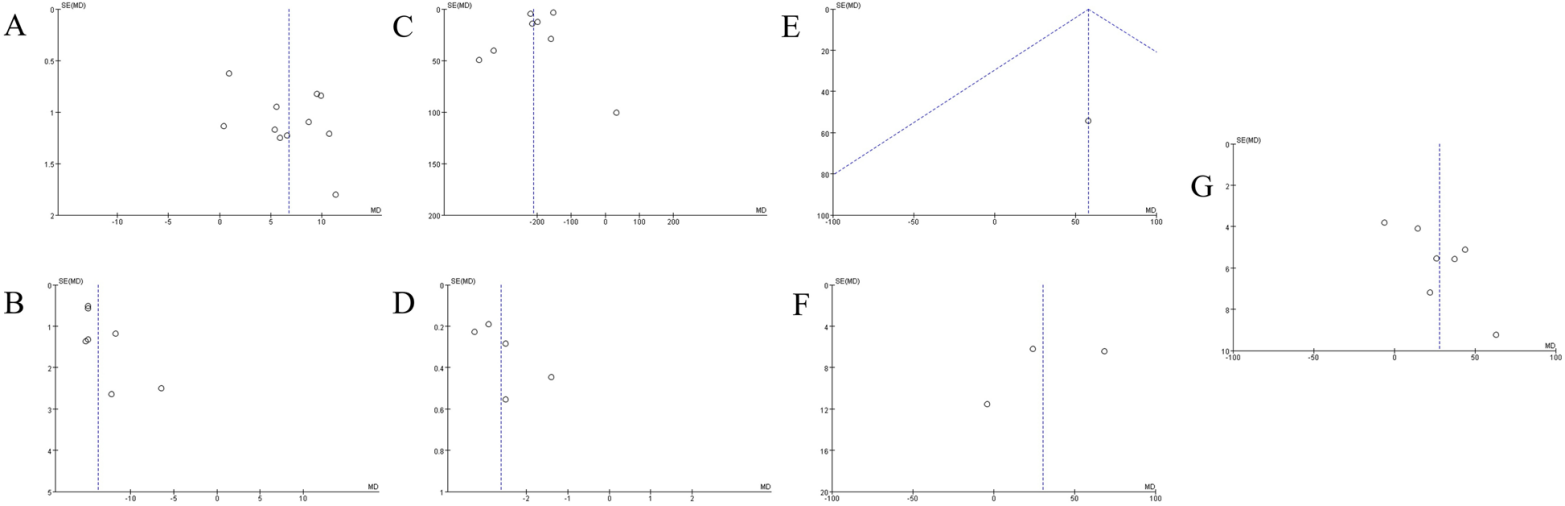
The funnel plot of each value of DU patients before and after surgery for more than a year. **A** *Q*_max_, maximal flow rate on uroflowmetry. **B** IPSS, International Prostate Symptom Score. **C** PVR, post-void residual. **D** QoL, quality of life. **E** Voided volume. **F** BCI, bladder contractility index. **G** PdetQ_max_, maximal detrusor pressure at maximal flow rate

According to the funnel plots (Figs. 7 and 8), significant publication bias was found in relevant pooled results.

## Discussion

BPH is highly prevalent among older males. BPH patients usually suffer from DU. The efficacy of current medical treatment for BPH is unsatisfactory. When comes to surgical treatment, due to the presence of DU in some patients, the efficacy of surgery remains controversial. What is more, considering the high cost of surgery, and potential adverse effects, this therapeutic option is debatable. This article reviewed the studies regarding surgical treatment for BPH patients with DU. According to the results of our study, BPH patients with DU who underwent the surgical treatment showed significant improvement in terms of subjective symptoms and urodynamic screening indicators.

The strength and main contribution of the present study is that in the case that the effects of surgery for DU patients in various studies are controversial, the results from a systematic review, which is relatively fair and more acceptable, may be conducive to reaching a consensus in the field.

In our study, subgroup analysis wasn’t conducted for the specific surgical procedures, such as TURP, PVP, and TUIP, because of the lack of relevant original researches. Significant differences may exist in the efficacy, morbidity, resection completeness, duration of benefit, or other variables among these surgical modalities. Independent meta-analyses, head-to-head randomized controlled trials, and other comparative studies should be conducted in the future to directly compare TURP, PVP, and TUIP in treating BPH-DU patients. This granular assessment would better delineate particular advantages from specific surgical modalities, and could help shape guidelines and practice. Moreover, some other surgical modalities, such as HoLEP, are not included in this review due to invalid data or lack of relevant studies. Future investigation should include more surgical options to find appropriate surgical modalities for BPH patients with DU. At the same time, we noticed that some researches may use clean intermittent catheterization to treat LUTS symptoms [20], in which a significant improvement in bladder accommodation was observed. However, it may cause urinary tract infections or other complications [21], and its high frequency of use may also affect the QoL. The comparison between clean intermittent catheterization and surgery is needed in the future. Moreover, the side effects and postoperative complications were not assessed in enrolled studies, such as reduced stream, intermittent stream, hesitancy, straining, urgency, incomplete emptying, and urge incontinence, which prevented the determination of comprehensive risk–benefit ratios to inform surgical decision-making. Subsequent studies should rigorously track and report the incidence of adverse events like infection, bleeding, and erectile dysfunction, which may attenuate the benefits achieved in symptomatic or urodynamic improvements. Similarly, several included studies only achieved up to 12 months of postoperative follow-up, which limits the analysis of durability of effects and may bring bias. Pragmatic and longitudinal studies with a minimum 5–10-year follow-up, ideally lifetime retrospective cohorts, would more persuasively demonstrate lasting gains in voiding function, flow metrics, and patient symptoms, rather than transient improvements from surgery. What’s more, BPH/LUTS prevalence estimates are infrequently reported by race/ethnicity. Due to the lack of information regarding the races of participants in enrolled studies, we cannot conduct subgroup analyses regarding the races. We expect that future articles will evaluate the role of racial disparities in the efficacy of surgical treatment. In terms of medication therapy, only two included articles compared surgical treatment with medication therapy, which significantly restricted comparable claims of increased efficacy over conventional drug regimens in BPH-DU. More comparative trials between transurethral methods and medicinal therapy, as well as cost-effectiveness studies, are needed to assess symptom benefits against procedure costs and morbidities.

One of the included articles classified the degree of symptoms of patients [19]. The results indicated that patients with mild and/or moderate symptoms had better surgical outcomes and QoL improvement than patients with severe DU. However, considering potential complications or risk of sequelae, surgical treatment conferred more benefits even in cases with severe DU compared to the other treatment methods (PdetQ_max_ 37.2 ± 29.3 vs 15.2 ± 4.4, *P* < 0.05; *Q*_max_ 8.3 ± 4.4 vs 2.9 ± 2.0, *P* < 0.05; PVR 157.3 ± 86.9 vs 316.7 ± 82.2, *P* < 0.05; IPSS 16.1 ± 6.9 vs 28.3 ± 8.4, *P* < 0.05). This study indicated the need for a more detailed delineation of the DU patient. Appropriate treatment methods for patients with different degrees of DU should be identified clearly. However, most relevant clinical studies did not classify patients based on their specific symptoms, which posed obstacles to our further analysis. Future trials should utilize strict selection criteria, and subgroup analyses adjusting for clinical factors should be conducted, particularly regarding mild, moderate, and severe DU grades, which may exhibit differing surgical suitability.

In our study, the results for the comparison between before surgery and after surgery within half a year were instability. The potential reasons included the variations in surgery type, limited sample size, the complexity, and heterogeneity of these patients, or their non-standardized management.

Although we collected IPSS, which included the self-reported QoL, and other objective indicators, patient perceptions, such as detailed assessment of QoL and specific voiding efficiency metrics, such as bladder contractility, were significantly underreported. Comprehensive prospective studies that focus on the comprehensive capture of subjective symptom scores, uroflow dynamics, voiding diaries, and adverse events would give more patient-centered evidence to guide care.

In addition, our study has some other limitations. First of all, due to the lack of results from multivariate analysis, the pooled results in our study didn’t adjust for covariates, which may bring bias. However, the strict selection criteria and clear definition of detrusor underactivity in some studies (Supplementary Table 2) may help reduce the bias caused by the lack of adjustment of confounding factors. Second, clinical or methodological differences among the original papers brought significant variability. The random-effects model, which is known to generate more conservative findings, was used to reduce this impact. Although we tried to contact with study authors to identify additional studies, we did not receive a reply or eligible data. Finally, there are relatively few studies comparing patients with or without surgery. Such studies can give us a more direct understanding of the results of the surgery.

## Conclusions

Our meta-analysis indicates that transurethral surgical treatment can improve the patient's symptoms. Within half a year, the *Q*_max_, voided volume, BCI, PdetQ_max_, IPSS, and QoL of DU patients showed great improvement. Even after more than a year, significant improvement remains.

### Supplementary Information

**Additional file 1: Supplementary Table 1. **Quality assessment of Cohort studies by Newcastle–Ottawa Scale. **Supplementary Table 2.** Exclusion criteria and definition of DU of the eligible studies.

## Data Availability

The data underlying this article are available in the article and in its online supplementary material.

## References

[1] Chapple CR, Wein AJ, Abrams P, Dmochowski RR, Giuliano F, Kaplan SA (2008). Lower urinary tract symptoms revisited: a broader clinical perspective. Eur Urol.

[2] Osman NI, Chapple CR, Abrams P, Dmochowski R, Haab F, Nitti V (2014). Detrusor underactivity and the underactive bladder: a new clinical entity? A review of current terminology, definitions, epidemiology, aetiology, and diagnosis. Eur Urol.

[3] Langan RC (2019). Benign Prostatic Hyperplasia. Prim Care.

[4] Seki N, Yuki K, Takei M, Yamaguchi A, Naito S (2009). Analysis of the prognostic factors for overactive bladder symptoms following surgical treatment in patients with benign prostatic obstruction. Neurourol Urodyn.

[5] Monoski MA, Gonzalez RR, Sandhu JS, Reddy B, Te AE (2006). Urodynamic predictors of outcomes with photoselective laser vaporization prostatectomy in patients with benign prostatic hyperplasia and preoperative retention. Urology.

[6] Miernik A, Gratzke C (2020). Current Treatment for Benign Prostatic Hyperplasia. Dtsch Arztebl Int.

[7] Stroup DF, Berlin JA, Morton SC, Olkin I (2000). Williamson GD, Rennie D, et al. Meta-analysis of observational studies in epidemiology: a proposal for reporting. Meta-analysis Of Observational Studies in Epidemiology (MOOSE) group.. Jama.

[8] Liberati A, Altman DG, Tetzlaff J, Mulrow C, Gøtzsche PC, Ioannidis JP (2009). The PRISMA statement for reporting systematic reviews and meta-analyses of studies that evaluate healthcare interventions: explanation and elaboration. BMJ.

[9] Ma LL, Wang YY, Yang ZH, Huang D, Weng H, Zeng XT (2020). Methodological quality (risk of bias) assessment tools for primary and secondary medical studies: what are they and which is better?. Mil Med Res.

[10] Choi SW, Choi YS, Bae WJ, Kim SJ, Cho HJ, Hong SH (2011). 120 W Greenlight HPS laser photoselective vaporization of the prostate for treatment of benign prostatic hyperplasia in men with detrusor underactivity. Korean J Urol.

[11] Lebani BR, Barcelos ADS, Gouveia D, Girotti ME, Remaille EP, Skaff M (2023). The role of transurethral resection of prostate (TURP) in patients with underactive bladder: 12 months follow-up in different grades of detrusor contractility. Prostate.

[12] Lee KH, Kuo HC (2019). Recovery of voiding efficiency and bladder function in male patients with non-neurogenic detrusor underactivity after transurethral bladder outlet surgery. Urology.

[13] Masumori N, Furuya R, Tanaka Y, Furuya S, Ogura H, Tsukamoto T (2010). The 12-year symptomatic outcome of transurethral resection of the prostate for patients with lower urinary tract symptoms suggestive of benign prostatic obstruction compared to the urodynamic findings before surgery. BJU Int.

[14] Rubilotta E, Balzarro M, Gubbiotti M, Antonelli A (2020). Outcomes of transurethral resection of the prostate in unobstructed patients with concomitant detrusor underactivity. Neurourol Urodyn.

[15] Sokhal AK, Sinha RJ, Purkait B, Singh V (2017). Transurethral resection of prostate in benign prostatic enlargement with underactive bladder: a retrospective outcome analysis. Urol Ann.

[16] Thomas AW, Cannon A, Bartlett E, Ellis-Jones J, Abrams P (2004). The natural history of lower urinary tract dysfunction in men: the influence of detrusor underactivity on the outcome after transurethral resection of the prostate with a minimum 10-year urodynamic follow-up. BJU Int.

[17] Thomas D, Zorn KC, Zaidi N, Chen SA, Zhang Y, Te A (2019). Does urodynamics predict voiding after benign prostatic hyperplasia surgery in patients with detrusor underactivity?. Asian J Urol.

[18] Wu SY, Kuo HC (2020). Predictive factors for recovery of voiding function after transurethral prostate surgery in men with small prostate volume and very low detrusor contractility. Low Urin Tract Symptoms.

[19] Yu Z, Li J, Li Z, Hou R (2015). Photoselective vaporization of the prostate and simultaneous suprapubic cystostomy for the treatment of benign prostatic hyperplasia in patients with mild to severe detrusor underactivity. Urol Int.

[20] Méndez-Rubio S, López-Pérez E, Laso-Martín S, Vírseda-Chamorro M, Salinas-Casado J, Esteban-Fuertes M (2020). The role of clean intermittent catheterization in the treatment for detrusor underactivity. Actas Urol Esp (Engl Ed).

[21] Wyndaele JJ, Brauner A, Geerlings SE, Bela K, Peter T, Bjerklund-Johanson TE (2012). Clean intermittent catheterization and urinary tract infection: review and guide for future research.. BJU Int.

