# Supplementary material for: Transurethral surgical treatment for benign prostatic hyperplasia with detrusor underactivity: a systematic review and meta-analysis
Source: Syst Rev. 2024 Mar 22;13:93. doi: 10.1186/s13643-024-02514-3 (PMC10958878; doi:10.1186/s13643-024-02514-3)
Supplement: Supplementary file 1 — Additional file 1: Supplementary Table 1. Quality assessment of Cohort studies by Newcastle–Ottawa Scale. Supplementary Table 2. Exclusion criteria and definition of DU of the eligible studies. [file 13643_2024_2514_MOESM1_ESM.docx]

Supplementary Table 1. Quality assessment of Cohort studies by Newcastle–Ottawa Scale.

| Included Study | Selection | | | | Comparability | | Outcome | | | Total Scores |
| --- | --- | --- | --- | --- | --- | --- | --- | --- | --- | --- |
|  | A | B | C | D | A1 | B1 | A2 | B2 | C2 |  |
| Thomas 2004 | 1 | 1 | 1 | 0 | 1 | 1 | 1 | 1 | 0 | 7 |
| Masumori 2010 | 1 | 1 | 1 | 0 | 1 | 1 | 1 | 1 | 0 | 7 |
| Choi 2011 | 1 | 1 | 1 | 0 | 1 | 1 | 1 | 1 | 1 | 8 |
| Yu 2015 | 1 | 1 | 1 | 0 | 1 | 1 | 1 | 1 | 1 | 8 |
| Sokhal 2017 | 1 | 1 | 1 | 0 | 1 | 1 | 1 | 1 | 1 | 8 |
| Lee 2019 | 1 | 1 | 1 | 0 | 1 | 1 | 1 | 1 | 1 | 8 |
| Thomas 2019 | 1 | 1 | 1 | 0 | 1 | 1 | 1 | 1 | 1 | 8 |
| Rubilotta 2020 | 1 | 1 | 1 | 1 | 1 | 1 | 1 | 1 | 1 | 9 |
| Wu 2020 | 1 | 1 | 1 | 0 | 1 | 1 | 1 | 1 | 1 | 8 |
| Lebani | 1 | 1 | 1 | 1 | 1 | 1 | 1 | 1 | 1 | 9 |

A: Representativeness of exposed cohort. B: Representativeness of unexposed cohort. C: Ascertainment of exposure (If the exposure data was obtained from prescription database or medical record). D: Outcome was not present at start. A1: Important factor. B1: Additional factor. A2: Assessment of outcome. B2: Exposure Follow-up for outcomes. C2: Rate of follow-up

Supplementary Table 2. Exclusion criteria and definition of DU of the eligible studies

| Study | Exclusion criteria | Definition of DU |
| --- | --- | --- |
| Thomas 2004 | N/A | Q_max_ (PdetQ_max_) of < 40 cmH_2_O, with a Q _max_ of < 15 mL/s |
| Masumori 2010 | Patients with prostate cancer, neurological diseases and the impossibility of symptomatic examination | N/A |
| Choi 2011 | N/A | N/A |
| Yu 2015 | Patients with neurogenic bladder;  Patients with BOO due to causes other than BPH;  Patients with a history of previous prostate or urethral surgery;  Patients who surgically confirmed prostate cancer  Patients with bladder tumors or stone disease | Q_max_ (Pdet_max_) of <40 cm H_2_O;  Wave-like detrusor contraction with a Q max of <10 ml/s |
| Sokhal 2017 | Patients with history of congenital or acquired neurologic conditions, neurogenic bladder, spinal trauma or surgery, pelvic trauma or surgery, diabetes mellitus with end organ damage, urethral pathology or surgery, and prostatic cancer | N/A |
| Lee 2017 | N/A | urodynamic evidence of low detrusor pressure (<40 cm H_2_O), low flow rate (<10 mL/s), a postvoid residual urine volume >300 mL, and a voiding efficiency (VE) of <33% |
| Thomas 2019 | Patients with repeat surgeries or prostate cancer, urologic injury, pelvic surgery or radiation | low detrusor contractions (< 30 cmH_2_O) or no contraction during the emptying phase |
| Rubilotta 2019 | Patients with neurological diseases, previous TURP, previous pelvic surgery/irradiation, previous interventions on the urethra | BCI weak category (≤100); concomitant Schäfer nomograms contractility classes very weak/weak |
| Wu 2020 | N/A | low detrusor voiding pressure (PdetQ_max_ < 40 cmH_2_O), low maximum flow rate (Q_max_ < 10 mL/s), large PVR (> 300 mL), and a low voiding efficiency (VE < 33%) |
| Lebani 2023 | Patients with suspicious or diagnosed prostate cancer, urethral stenosis and neurological diseases | N/A |

Q_max_, maximal flow rate on uroflowmetry, PVR, post-void residual; BCI, bladder contractility index, PdetQ_max_, maximal detrusor pressure at maximal flow rate,

VE, voiding efficiency
